# Bipolar clavicular fractures and treatment options

**DOI:** 10.1007/s00068-019-01191-5

**Published:** 2019-08-06

**Authors:** Kristian J. de Ruiter, Tjibbe J. Gardenbroek, Kelly Bos, Mark van Heijl, Jens A. Halm

**Affiliations:** Trauma Unit, Department of Surgery, Amsterdam University Medical Centres, Amsterdam, The Netherlands

**Keywords:** Bipolar, Clavicle, Fracture, Review

## Abstract

**Introduction:**

Fractures of the clavicle are common injuries, accounting 2.6–4% of all fractures in adults. Of these fractures, 21–28% are lateral clavicle fractures and 2–3% are medial clavicle fractures. Bipolar clavicle fractures are defined as a lateral and medial fracture and are uncommon. There is no consensus on the treatment of these fractures. The aim of this study is to provide a treatment on bipolar clavicle fractures based on the current literature.

**Methods:**

The electronic databases PubMed, the Cochrane library and EMBASE were searched up on September 25th, 2017. Two reviewers (KR and TG) independently screened titles and abstracts for their relevance. Studies designed to evaluate the outcomes of conservative and/or operative treatment of segmental bipolar clavicle fractures in adults (> 16 years) were included. Editorials and commentaries were excluded, as well as synthetic, cadaveric and animal studies. Primary outcomes considered were pain reduction and shoulder function. Secondary outcomes considered are complications.

**Results:**

Ten studies reporting results from ten patients were included for the review. In most patients, if treated operatively, surgical treatment with the use of double plating was performed. Only in elderly patients conservative treatment was adopted. All included patients were pain free and had a full range of motion after 3–6 months. Only two case reports provided a DASH score, while in eight studies no functional outcome score was measured.

**Conclusion:**

A missed bipolar fracture can complicate the clinical progress. Surgical management of these fractures may be necessary; however, the treatment of choice depends on the age of the patient, daily activities and comorbidity.

**Electronic supplementary material:**

The online version of this article (10.1007/s00068-019-01191-5) contains supplementary material, which is available to authorized users.

## Introduction

Fractures of the clavicle are common injuries, accounting 2.6–4% of all fractures in adults. Of these fractures, 21–28% are lateral clavicle fractures and 2–3% are medial clavicle fractures [1, 2]. Bipolar clavicle fractures are defined as a lateral and medial clavicle fracture; on the other hand, the term ‘floating clavicle’ refers to a sternoclavicular and an acromioclavicular luxation, also known as a panclavicular dislocation [3–13]. This study describes a patient with a bipolar clavicle fracture and presents an overview of the current literature.

## Case description

A 23-year-old man was admitted after a high speed motor vehicle accident. A total body computed tomography was performed at the emergency department and showed the following injuries: cervical 4–6 fractures with spinal cord injury, left vertebral artery dissection, left transverse process fractures thoracic 5–8, fractures of ribs 1 and 2 on the left side, bilateral contusions of the lung and a bipolar clavicle fracture on the left side. The patient was admitted to our institution for an ASIA C type injury following local spinal injury protocol. The patient remained hemodynamically stable, but became symptomatic for pain of the left clavicle with a median NRS of 5 during the admission, measured three times a day.

Additional X-rays showed a medial clavicle fracture with 15 mm displacement and a lateral fracture with 10 mm displacement (IMAGE 1 in Supplementary material). Fracture fixation was performed by double plating after fracture reduction, utilizing a Depuy Synthes 2.7–3.5 mm Variable Angle (VA) Locking Compression Plate (LCP) placed anteriorly at the medial fracture site and a Depuy Synthes superior 2.7/3.5 LCP clavicle plate with lateral extension was placed at the lateral fracture site (IMAGE 2 in Supplementary material). Postoperative X-rays showed an anatomical reduction (IMAGE 3 in Supplementary material). Direct post-operative the patient reported significant pain relief with a NRS of 0–2 and started active range of motion of the shoulder with help of a physiotherapist. Weight bearing activities were avoided for 6 weeks. The patient was discharged to a rehabilitation centre for further treatment of his spinal cord injury and bipolar clavicle fracture. The patient visited the outpatient clinic after 6 weeks. X-Rays showed initial signs of consolidation and a maintained anatomical reduction (IMAGE4 in Supplementary material). Range of motion: abduction/adduction: 65/0/20 degrees. Pain was reduced to a NRS of 1. X-Ray in July 2019 showed complete consolidation, no implant failures (IMAGE5 in Supplementary material).

## Literature review

The electronic databases PubMed, the Cochrane library and EMBASE were searched up on September 25th, 2017. The search was performed with both keywords and MeSH terms. The search consisted of: bipolar OR segmental AND clavicle. Two reviewers (KR and TG) independently screened titles and abstracts for their relevance. Additionally, the reference lists of all included articles were additionally searched for other relevant references.

Studies designed to evaluate the outcomes of conservative and/or operative treatment of segmental bipolar clavicle fractures in adults (> 16 years) were included. Editorials and commentaries were excluded, as well as synthetic, cadaveric and animal studies. Inclusion was not otherwise restricted by study size, language or publication type.

Primary outcomes considered were pain reduction and shoulder function. Secondary outcomes considered are complications, such as infection, non-union and implant failure. None of the ten case reports described how pain, shoulder function and complications were scored. Only two case reports provided a DASH score.

## Results

**Fig. 1:**
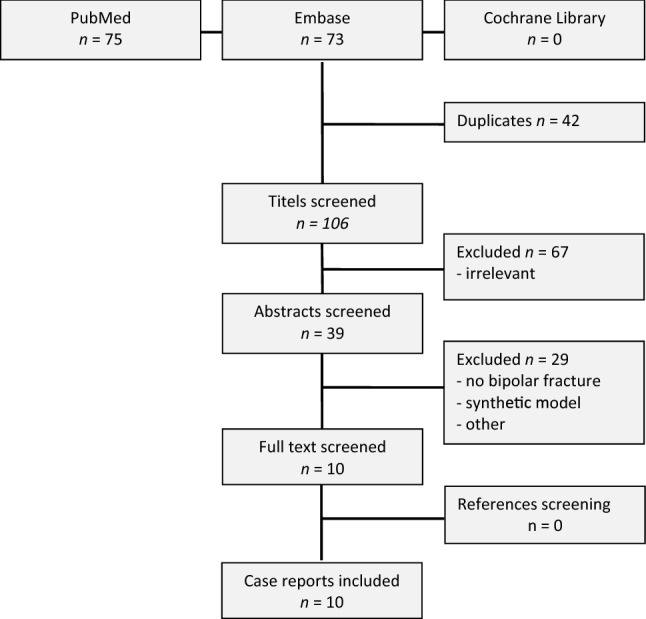
Flow chart of article inclusion

Details of the literature search are shown in Fig. 1. All ten included studies are single case reports. Characteristics of the studies are shown in Table 1. Three elderly patients were treated conservatively and seven patients were treated operatively. In most of these operatively treated patients double plate fixation was performed. In two cases clinical impairment was seen. The medial clavicle fracture was missed directly after the injury. In the first case the medial fracture was found after 4 days and before surgery, so no extra operation was needed. In the second case the medial fracture was found after 28 days. An extra operation was performed. In all cases the fractures healed and in one case the operation was complicated by a pneumothorax [8]. In three cases implant removal was performed.

**Table 1:** Characteristics of included studies

References	Year	Study design	Gender	Age (years)	Trauma	Dislocation	Treatment	Method	Postoperative treatment	Result
Talboys et al. [3]	2016	Case report	Female	79	Stumbled over slipper	Medial dislocation	Conservative	Sling	–	Pain free FROM after 3 months
Yalizis et al. [4]	2016	Case report	Male	38	Fall from push bike	Lateral and medial dislocation	Surgical	Lateral hook plate, medial plate	–	Pain free FROM after 3 months
Grossi [5]	2015	Case report	Male	41	Fall from roof	Lateral and medial dislocation	Surgical	Superior reconstruction plate and lateral K-wire	7 weeks no weight-bearing + physiotherapy	Pain free FROM after 3.5 months
Sopu et al. [6]	2015	Case report	Male	52	Fall from push bike	Medial dislocation	Surgical	Medial plate, lateral no fixation.	6 weeks no weight-bearing	Pain free FROM after 4 months
Varelas et al. [7]	2015	Case report	Female	68	Fall on ice	Lateral and medial dislocation	Surgical	Medial and lateral locking plate	Sling, no weight-bearing for 6 weeks	Pain free FROM after 3 months. DASH = 5
Skedros et al. [8]	2014	Case report	Male	33	Motocross accident	Lateral and medial dislocation	Surgical	Medial and lateral reconstruction plate	12 weeks no weight-bearing + physiotherapy	Pain free FROM after 5 months. DASH = 8
Sethi et al. [9]	2012	Case report	Female	70	Fall from stairs	Medial and lateral undisplaced	Conservative	Sling	4 weeks immobilisation in sling	Pain free FROM after 6 months
Miller et al. [10]	2009	Case report	Male	17	Car accident	Lateral and medial dislocation	Surgical	Medial and lateral locking plate	–	Pain free FROM after 6 months
Heywood et al. [11]	2005	Case report	Male	54	Molest	Lateral and medial dislocation	Surgical	Lateral hook plate, medial plate	Physiotherapy	Consolidation after 3 months
Pang et al. [12]	2003	Case report	Male	76	Motorcycle accident	Medial dislocation	Conservative	Sling	–	FROM after 6 months with no instability

*FROM* functional range of motion

## Discussion

This study describes the treatment of a patient with a bipolar clavicle fracture and presents a review of the current literature regarding bipolar clavicle fractures. Only ten single case reports could be included in the review, describing varying treatment modalities. In most patients, surgical treatment with the use of double plating was performed. Only in elderly patients conservative treatment was adopted. All included patients were pain free and had a full range of motion after 3–6 months. Furthermore only 2 of the 10 available case reports described a validated functional outcome score.

Most of the bipolar fractures are sustained by high energy trauma. Of all clavicle fractures 21–28% are lateral fractures and 2–3% are medial fractures [1]. A truly bipolar clavicle fracture is a rare entity. The term ‘’floating clavicle’’ refers to an AC- and SC-displacement without fractures [13, 14]. A bipolar fracture can easily be missed and may therefore complicate the clinical progress. It is advised to assess the whole length of the clavicle clinically and radiologically when fractured, in order not to miss a bipolar fracture. When there is any doubt, the use of a computed tomography scan is recommended.

Research has shown that a non-operative treatment of an isolated, non-displaced, medial clavicle produced limited pain and excellent functional outcomes [2]. Displaced medial fracture require operative treatment [15].

Lateral fractures require operative management if displaced [16]. Two studies in this review used a hook plate for fixation of the lateral fracture. One article has shown that locking plate fixation is superior compared to hook plate fixation in lateral clavicle fractures [17].

This study presents a complete overview of the current literature. Due to the rarity of this injury, a formal meta-analysis of the literature was not possible.

These injuries may impair clinical progress if undetected, as seen in two cases in our review. We suggest that operative treatment should be performed for displaced medial and lateral fractures in a bipolar fracture, although the treatment of choice depends on the age of the patient, daily activities and comorbidity. While displaced fractures may benefit from surgery, treating only one part of a bipolar clavicle fracture may complicate treatment should the other part become displaced at a later instance.

## Conclusion

Based on the current evidence, no strong recommendation can be made on the treatment of bipolar clavicle fractures. Literature has shown that operative treatment should be performed in isolated medial an lateral clavicle fractures. We suggest that displaced bipolar clavicle fractures should be treated operatively. However the treatment of a bipolar clavicle fracture depends on the age of the patient, daily activities and comorbidity.

## Electronic supplementary material

Below is the link to the electronic supplementary material.

Supplementary material 1 (DOC 203 kb)

Supplementary material 2 (DOC 421 kb)

Supplementary material 3 (DOC 79 kb)

Supplementary material 4 (DOC 37 kb)

Supplementary material 5 (DOCX 242 kb)
