# Supplementary figures and images for: Bipolar clavicular fractures and treatment options
Source: Eur J Trauma Emerg Surg. 2019 Aug 6;47(5):1407–10. doi: 10.1007/s00068-019-01191-5 (PMC8476377; doi:10.1007/s00068-019-01191-5)

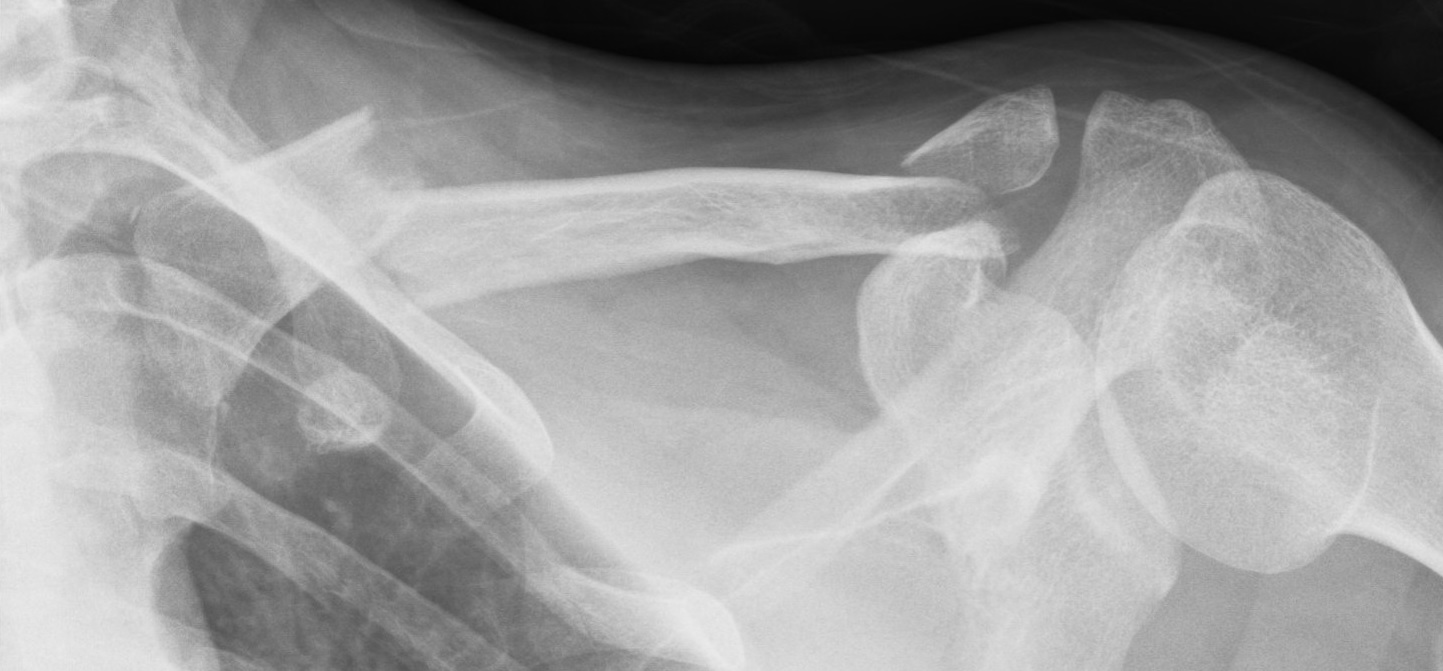
**Image 1**

Supplement: Supplementary file 1 — Supplementary material 1 (DOC 203 kb) [file 68_2019_1191_MOESM1_ESM.doc]

**Image2**


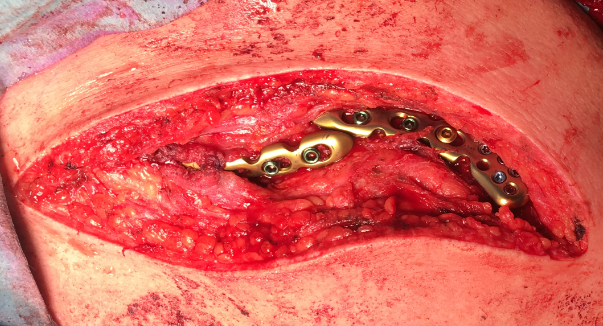

Supplement: Supplementary file 2 — Supplementary material 2 (DOC 421 kb) [file 68_2019_1191_MOESM2_ESM.doc]

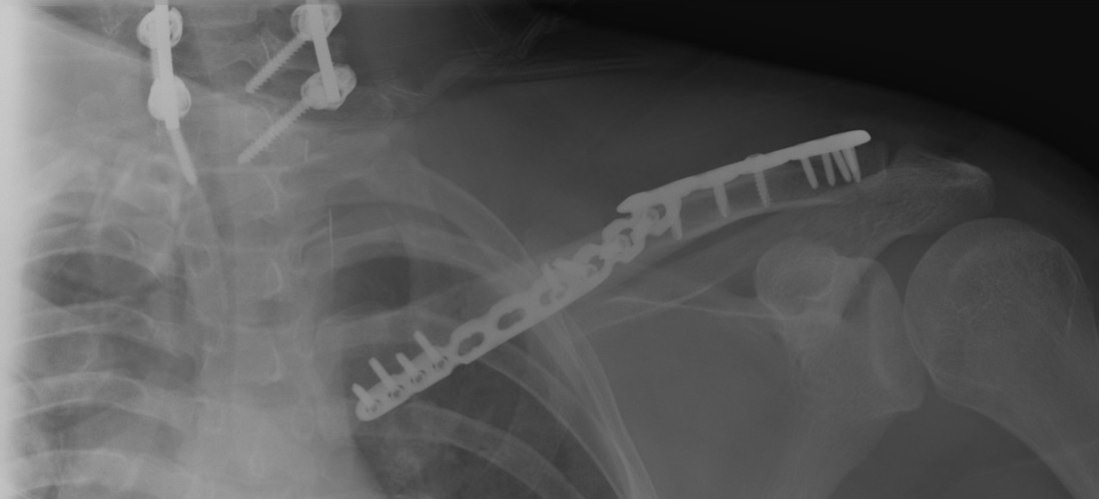
**Image 3**

Supplement: Supplementary file 3 — Supplementary material 3 (DOC 79 kb) [file 68_2019_1191_MOESM3_ESM.doc]

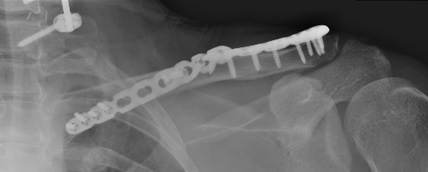
**Image 4**

Supplement: Supplementary file 4 — Supplementary material 4 (DOC 37 kb) [file 68_2019_1191_MOESM4_ESM.doc]

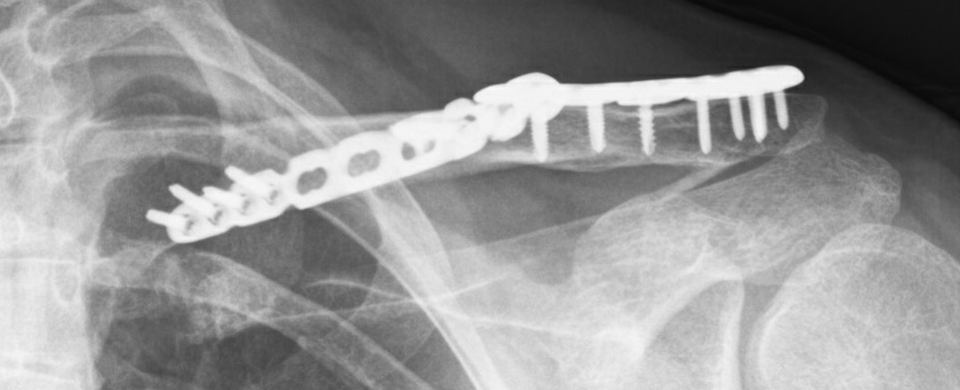
**Image 5**

Supplement: Supplementary file 5 — Supplementary material 5 (DOCX 242 kb) [file 68_2019_1191_MOESM5_ESM.docx]
